# May-Thurner Syndrome with Large Abdominal Varicosity, Treated Successfully Using Multiple Approaches

**DOI:** 10.1155/2019/7079307

**Published:** 2019-04-30

**Authors:** Lori Jia, Jason Alexander, Nedaa Skeik

**Affiliations:** Minneapolis Heart Institute, Minneapolis, MN, USA

## Abstract

May-Thurner syndrome (MTS) is a venous outflow obstruction disorder characterized by compression of the left common iliac vein by an overriding right common iliac artery. MTS primarily affects young to middle-aged women, although many patients remain entirely asymptomatic. Anatomic variations of MTS, while uncommon, have been described. Treatment usually involves endovascular management, including thrombolysis and/or thrombectomy with or without inferior vena cava filter placement, followed by angioplasty and stenting of the left common iliac vein. We report a unique case of a 31-year-old woman who presented with MTS-related deep vein thrombosis accompanied by symptomatic abdominal and pelvic varicosities. The varicosities were treated successfully using multiple procedures, resulting in complete resolution of all symptoms. Our case discusses a treatment approach for an unusual presentation of MTS-related postthrombotic syndrome, and provides a brief literature review of MTS complications and management.

## 1. Case Report

**Figure 1:**
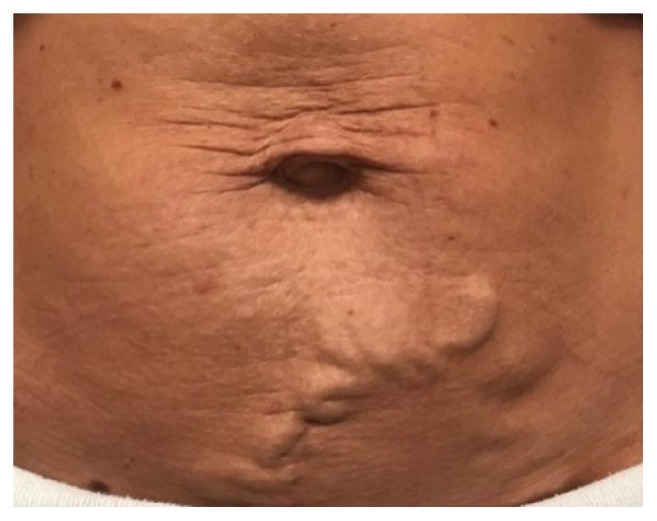
Patient presented with large tortuous varicosities at the lower abdomen and left groin.

**Figure 2:**
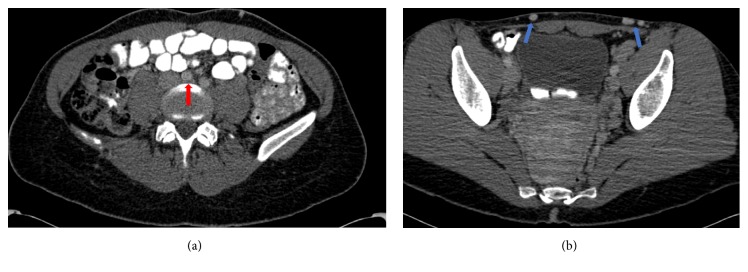
CT venogram of the abdomen pelvis revealed (a) compression of the left common iliac vein by the right common iliac artery (red arrow), and (b) abdominal and left groin varicosities (blue arrows).

A 31-year-old woman with a history of left common iliac vein thrombosis presented with symptomatic lower abdominal and left groin superficial varicosities associated with itching, swelling, and discomfort. Vital signs revealed a blood pressure of 114/80 mmHg and pulse of 72 beats/minute. Physical examination confirmed the presence of large tortuous varicosities at the lower abdomen and left groin (Figure 1). The patient had previously been diagnosed with left common iliac vein thrombosis during her first pregnancy, 10 years earlier, that was only managed with enoxaparin injections. Over the next few years, she developed lower abdominal and left groin varicosities that worsened significantly during her second and third pregnancies without confirmed recurrent deep vein thrombosis (DVT). The patient denied leg swelling prior to the DVT event. She also denied any history of abdominal trauma, other thromboembolic events, or family history of vascular anomalies. Computed tomography (CT) venography revealed compression of the left common iliac vein by the right common iliac artery without evidence of acute thrombosis, indicating a diagnosis of May-Thurner syndrome- (MTS-) related anatomy (Figure 2).

**Figure 3:**
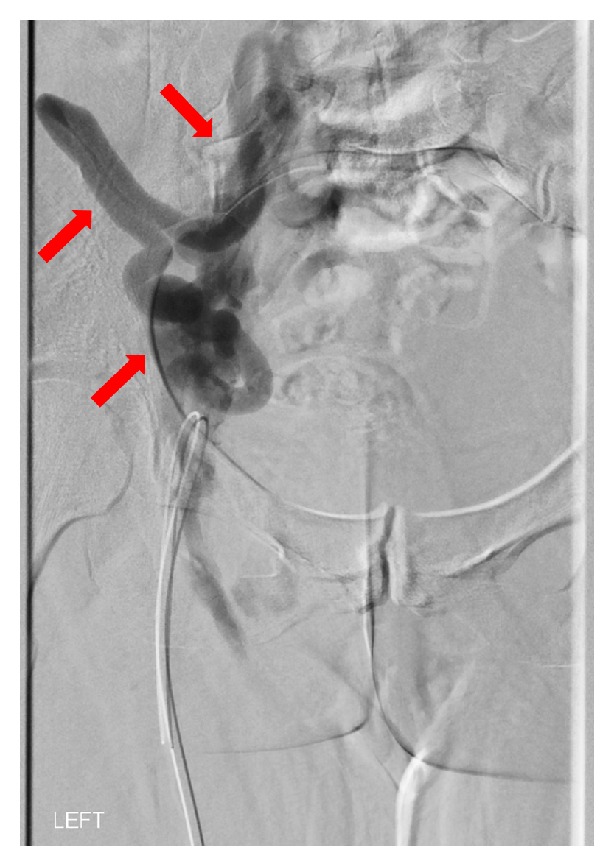
Catheter-based venogram confirmed May-Thurner anatomy with sequelae of chronic DVT in the left iliac vein and cross-pelvic drainage via pelvic and abdominal wall varices (red arrows).

Catheter-based venography with hemodynamic pressure measurements confirmed May-Thurner anatomy with sequelae of chronic DVT in the left iliac vein and cross-pelvic drainage via pelvic and abdominal wall varices (Figure 3). Successful recanalization was performed using percutaneous transluminal angioplasty with stenting of the left common iliac vein (20 mm x 55 mm Wallstent) and left external iliac vein (14 mm x 60 mm Protege). The patient was managed with apixaban (5 mg twice daily) for three months and clopidogrel (75 mg daily) for one month that was changed to aspirin (81 mg daily) in the long term. A 3-month follow-up CT venogram indicated a patent left iliac vein stent. However, the patient continued to present with painful, though slightly improved, lower abdominal and left groin varicosities.

**Figure 4:**
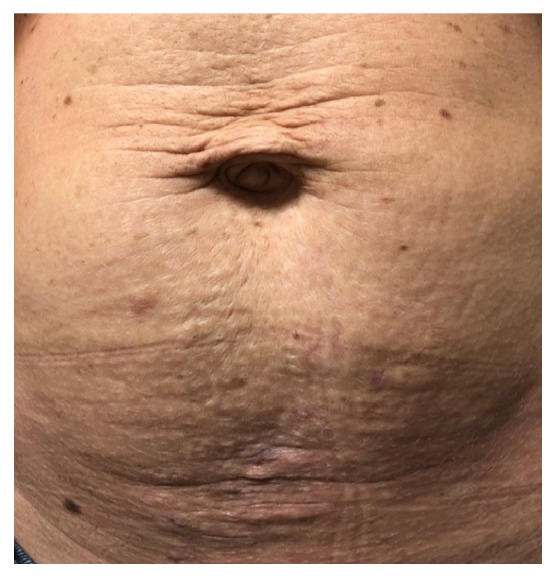
Successful stab phlebectomy and ultrasound-guided sclerotherapy resulted in complete resolution of the symptomatic varicosities.

Given these persisting symptoms, we performed successful stab phlebectomy of the large superficial abdominal varicosity. We also treated the deeper feeding branch and groin varicosities with ultrasound-guided sclerotherapy using a sclerosing foam (two injections of 1 cc of 3% sotradecol mixed with 2 cc of room air). The procedure resulted in complete resolution of the symptomatic lower abdominal and left groin varicosities (Figure 4). The patient did very well at 6- and 12-month follow-up visits while on aspirin, and a repeat CT venogram indicated a patent left iliac vein stent.

## 2. Discussion

May-Thurner syndrome (MTS) is a venous outflow obstruction disorder characterized by compression of the left common iliac vein by an overriding right common iliac artery [1]. The condition was first described in 1957, when May and Thurner discovered that 22% of 430 cadavers possessed this unique anatomic substrate [1]. A more recent retrospective analysis has supported this anatomic incidence, having identified significant (i.e., >50%) left common iliac vein compression in 24% of asymptomatic individuals [2]. However, the clinical incidence of MTS is relatively uncommon, reportedly occurring in only 2%-5% of patients who present with lower extremity venous disorder [3, 4]. This discrepancy between anatomic and clinical incidence thus demonstrates that left common iliac vein compression is necessary, but not sufficient, to cause symptomatic MTS [4].

At the same time, it is also likely that MTS-related DVT may be underreported. MTS primarily affects young to middle-aged women, whose presentations are often accompanied by a history of multiple pregnancies, the postpartum period, and oral contraceptive use [3, 5]. These more common causes of DVT can obscure the precise anatomic finding of MTS by deterring further workup after a confirmed DVT diagnosis [6]. The fact that there is a 55.9% predominance for left-sided DVT may also support the idea of an underreported prevalence [7]. An accurate MTS diagnosis is crucial, as failure to correct the anatomic substrate can lead to DVT recurrence, postthrombotic syndrome, and additional complications, including pulmonary emboli, chronic vein stasis, and iliac vein rupture [3, 6, 8, 9].

MTS presentation has two common variants. The more common variant involves DVT either provoked (i.e., with a discernible etiology) or unprovoked [10]. The less common variant involves progressive pain, unilateral left leg edema, varicose veins, and venous ulcers without antecedent acute thrombosis [10]. At the anatomic level, MTS is characterized by compression of the left iliac vein by the right iliac artery, although variations have been reported. These include compression of the right iliac vein by the left iliac artery; compression of the inferior vena cava by the right iliac artery; and concurrent compression of the left iliac vein, right iliac vein, and inferior vena cava by the right iliac artery [11–13]. Extensive pelvic collateral venous drainage is an additional hallmark of MTS [14, 15]. However, many patients with May-Thurner anatomy remain entirely asymptomatic and may only become symptomatic upon trauma or major surgery [2, 3, 5, 13].

A proper diagnosis of MTS should include imaging demonstration of pelvic venous compression and venous collateralization, as well as clinical manifestations such as DVT, leg edema, and varicose veins. Collateral veins often form in the pelvis, the majority of which emerge from the ipsilateral internal iliac vein, through the parametrial and presacral plexus, to the contralateral internal iliac vein [16]. Alternative routes can form through the ovarian vein [16]. Given the significant impact pregnancy imposes onto women's vascular system, pregnant women are especially prone to venous collateralization [17]. Based on our literature review, we found only two cases of pregnant women reported to have pelvic collaterals that manifest as suprapubic varicosities [17].

Computed tomography venography and magnetic resonance venography are two newer, minimally invasive modalities for evaluating venous architecture and estimating the degree of stenosis; however, cost, contrast, and availability are often significant considerations [3, 10, 18, 19]. Venography can help identify three common angiographic patterns in MTS [20]. These include focal stenosis or collateralized short-segment occlusion of the left common iliac vein, acute iliofemoral venous thrombosis with the underlying lesion revealed after successful thrombolysis, and chronic isolated thrombosis of the left common and external iliac veins with collaterals arising from the common femoral vein [20]. Intravascular ultrasonography (IVUS) and hemodynamic pressure measurements are also useful for confirming an MTS diagnosis [3]. In particular, IVUS has been recognized for its invaluable role in stent selection, deployment, and evaluation [3, 4].

Today, MTS patients rarely undergo highly invasive treatment [4]. Whereas surgical techniques have been associated with high morbidity and mixed patency rates, endovascular techniques have found great success with few operative risks [21–24]. Endovascular management often begins with venography and IVUS, which help confirm MTS and identify the degree of stenosis and pelvic venous collaterals. Based on the findings, thrombolysis and/or thrombectomy can be performed with or without inferior vena cava filter placement, followed by angioplasty and stenting of the left iliac vein [3, 4]. Percutaneous transluminal angioplasty without subsequent stent placement has yielded low patency rates, which suggests that the chronic compression of MTS cannot be relieved by temporary balloon angioplasty [25]. As a result, MTS treatment almost always requires high radial force stents [26]. A number of studies have demonstrated the safety and efficacy of catheter-directed thrombolysis and subsequent stent placement, with high rates of initial technical success as well as one-year patency rates [5, 25, 27].

Prior to addressing our patient's lower abdominal and left groin varicosities, we decided to first correct the underlying pathology by recanalizing and stenting the left iliac vein. Treating the collateral veins prior to correcting the underlying vein compression could have resulted in worse symptoms and outcome. Since the patient's varicosities did not completely resolve, and after continued iliac vein stent patency had been confirmed, we performed successful stab phlebectomy of the large abdominal varicosity and ultrasound-guided sclerotherapy of the feeding vein and left groin varicosities. We recommend a thoughtful approach in similar cases of collateral varicosities.

Following intervention, MTS patients are usually placed on a 4- to 6-week regimen of aspirin (81-325 mg daily) and clopidogrel (75 mg daily) to prevent stent thrombosis, after which they continue on one of the two antiplatelet agents indefinitely [10]. Patients who present with acute DVT and MTS are initiated on anticoagulation therapy for 3-6 months [10]. For patients with documented thrombophilia, long-term anticoagulation therapy may be considered [10]. There has been no consensus on postintervention antithrombotic therapy for MTS patients. Our current approach utilizes direct oral anticoagulant and single antiplatelet therapy for three months, before stopping anticoagulation and continuing aspirin (81 mg daily) on the long term if repeat imaging confirms stent patency.

Our case highlights the challenges of diagnosing MTS in young women during their child-bearing years. It also discusses a treatment approach for an unusual presentation of MTS-related postthrombotic syndrome.
